# Bronchial reactivity and asthma at school age after early-life metapneumovirus infection

**DOI:** 10.1183/23120541.00832-2023

**Published:** 2024-01-22

**Authors:** Åsne Myklebust, Melanie Rae Simpson, Jonas Valand, Vibeke Stenhaug Langaas, Tuomas Jartti, Henrik Døllner, Kari Risnes

**Affiliations:** 1 Department of Clinical and Molecular Medicine, Norwegian University of Science and Technology, Trondheim, Norway; 2 Children's Clinic, St Olavs Hospital, Trondheim University Hospital, Trondheim, Norway; 3 Department of Public Health and Nursing, Norwegian University of Science and Technology, Trondheim, Norway; 4 Department of Immunology and Transfusion Medicine, St Olavs Hospital, Trondheim University Hospital, Trondheim, Norway; 5 Research Unit of Clinical Medicine, University of Oulu, Oulu, Finland; 6 Department of Pediatrics and Adolescent Medicine, University of Oulu, Oulu, Finland; 7 Department of Pediatrics and Adolescent Medicine, Turku University Hospital and University of Turku, Turku, Finland

## Abstract

**Background:**

The association between early-life lower respiratory tract infection (LRTI) and asthma is well established. Knowledge about bronchial hyperresponsiveness (BHR) and asthma after metapneumovirus (MPV) LRTI is scarce. The aim of this study was to assess BHR and current asthma in school-aged children after hospital admission for early-life LRTI with MPV, and to compare with more well-known viruses, rhinovirus (RV) and respiratory syncytial virus (RSV), and with controls.

**Methods:**

A cohort consisting of children admitted for LRTI and controls was followed-up at school age with a clinical research assessment and lung function tests, including a methacholine provocation test. Current asthma was defined based on objective variable airway obstruction and clinical symptoms. BHR and asthma were compared according to viral groups.

**Results:**

135 children (median age 9.3 years) were included (16 MPV, 34 RV, 51 RSV, 13 mixed infections and 21 controls). Compared with controls there was increased BHR after MPV and RV LRTI (provocative dose causing a 20% fall in forced expiratory volume in 1 s and dose–response slope; p<0.05). Using Kaplan–Meier statistics, BHR was increased for MPV compared with both controls and RSV (p=0.02 and p=0.01). The proportion of children with current asthma at follow-up was higher in the LRTI children compared with the controls (46% versus 24%; p=0.06). Among children who had undergone MPV and RV infection, 50% fulfilled the asthma criteria compared with 43% in the RSV group (p=0.37).

**Conclusion:**

We found increased BHR and a high prevalence of asthma in school-aged children after early-life MPV infection, and findings were similar to RV, and less to RSV, compared with controls.

## Tweetable abstract

**Early-life lower respiratory tract infection with metapneumovirus, as well as more studied rhinovirus and RSV infections, is a risk factor for increased bronchial hyperreactivity and asthma in school-aged children** https://bit.ly/3U0pq3l

## Introduction

Asthma is the most common chronic disease in children, with a prevalence of 11% worldwide and 22% in high-income countries when including asthma-like symptoms [1–3]. Bronchial hyperresponsiveness (BHR) is an important feature of asthma; it is also associated with later asthma development [4, 5]. The association between early-life respiratory tract infections and asthma, and especially early-life lower respiratory tract infections (LRTIs), is well established [6, 7].

Early-life LRTIs represent a great burden to young children's health and ∼3% need hospitalisation [8, 9]. The most common aetiologies are respiratory syncytial virus (RSV) (30–76%), followed by rhinovirus (RV) (18–27%), thereafter metapneumovirus (MPV) (2–7%), coronaviruses (7%), human bocavirus (BoV) and influenza viruses, in addition to co-infections [10–12]. Risk for asthma development after early-life LRTI is strongly associated with a profile characterised by eczema, previous wheeze and RV [13, 14]. Considering viral aetiology alone, a systematic review has shown RV-induced bronchiolitis to be a nearly 3-fold stronger risk factor than RSV for pre-school wheeze and asthma [15].

Since MPV was discovered in 2001, the virus has been placed solidly among the viruses that cause LRTIs, but also asthma exacerbations [16–19]. MPV resembles RSV both genetically and regarding disease severity [20]. Despite this increasing knowledge on MPV's clinical and immunological profiles, long-term lung function including BHR and asthma after early-life MPV LRTI remains unexplored. Our present understanding of asthma pathogenesis implies an abnormal immune response to respiratory viruses, environmental agents or allergens that are responsible for the initiation and perpetuation of chronic inflammation in genetically susceptible individuals [6]. Modern asthma treatment approaches target these altered immune responses. Assessment of asthma development after different early-life viral infection exposures may contribute to understanding immune responses, and underpin prevention and treatment strategies, and we consider it both novel and important to describe long-term outcomes after MPV infection.

The aim of this study was to assess BHR and current asthma in school-aged children after hospital admission for early-life LRTI with MPV. For comparison we included corresponding groups after RV and RSV LRTI, and a control group with no LRTI during the first 2 years of life. We hypothesised that MPV-induced LRTI is associated with altered BHR and asthma risk, due to its close relation to RSV.

## Material and methods

### Study design and subjects

In this prospective study, we invited children from a Norwegian surveillance cohort of airway infections [21–23] to a clinical follow-up at school age. In addition to children with airway infection this cohort includes prospectively enrolled control children: elective surgery patients with no signs of airway infection were included for nasopharyngeal aspirates (NPAs) and clinical data collection, with the same methods as for the LRTI children. The selection criteria for follow-up for the LRTI children were all of the following: 1) hospitalisation for LRTI, 2) no parent-reported symptoms of previous LRTI, 3) age at exposure <2 years and 4) one or several of the following viruses: MPV, RV, RSV or BoV. They were classified into four virus groups: 1) MPV (inclusive viral co-detections other than RSV, RV or BoV), 2) RV only (single virus), 3) RSV only (single virus) and 4) mixed virus group. The control children were only eligible if they had no history of hospitalisation for LRTI nor asthma before 2 years of age. Also, all children invited for follow-up had reported no wheeze, asthma or any chronic disease (except from allergy and eczema) before the hospitalisation for LRTI (before 2 years of age for the controls). Parents were interviewed at follow-up and a full chart review was performed to assess the inclusion and exclusion criteria. The study was approved by the Regional Committee on Medical Research Ethics (REK 2016/540). Informed consent was given by the parents and collected at inclusion in early childhood, and a new informed consent was performed before the follow-up.

### Methods

#### Exposure

Clinical data and NPAs from hospitalisation were prospectively collected for both LRTI and control children (see supplementary material for details) [24].

#### Follow-up

The follow-up at school age was systematically set up for research and took place at the Research Facility Ward, St Olavs Hospital (Trondheim, Norway) between March 2017 and June 2019. Eligible children enrolled in the original cohort between 2006 and 2012 were identified and invited. The research visit was performed by a paediatric asthma specialist and a trained research nurse, both blinded to exposure. It included a systematic medical history, a clinical exam, and a blood sample for the analysis of leukocytes, differential blood count, total IgE and allergy panels for food and aeroallergies (see supplementary material for details). Lung function was measured with baseline spirometry and eligible children continued with a methacholine provocation test (MPT), followed by reversibility testing with salbutamol for all children (see supplementary material for details). A digital questionnaire for caregivers was based on the International Study of Asthma and Allergies in Childhood questionnaires [25].

#### Main outcomes

The main outcome of the study was the assessment of differences in BHR defined by PD_20_ values (see Definitions section). We also assessed two alternative measurements for BHR (by survival analyses and dose–response slope (DRS)) to assess the robustness of BHR differences. Due to limited power the assessment of current asthma prevalence was considered a secondary outcome. An additional secondary exploratory outcome was to assess the prevalence of current allergic asthma for different LRTI aetiologies compared with controls. We also explored the possible role of confounding by allergic history and pre-term birth on the association between viral exposure and current asthma.

#### Definitions

Variable airflow expiratory limitation, or reversibility, was defined as a fall in forced expiratory volume in 1 s (FEV_1_) of ≥20% during the MPT. For children not eligible for the MPT, reversibility was defined as an increase in FEV_1_ of ≥12% compared with baseline spirometry after salbutamol inhalation. PD_20_ was defined as the provocative dose (µg) of methacholine necessary to produce a fall in FEV_1_ of 20%. In the survival analysis, for children not eligible for the MPT, but with a positive reversibility test, PD_20_ was set to 1 µg for the purpose of statistical analyses. DRS (%·mg^−1^) was the ratio of maximum percentage decline of FEV_1_ (%) to the cumulative methacholine dose administered (mg).

Based on the Global Initiative for Asthma guidelines [3], current asthma was defined as: 1) presence of one or more typical asthma symptoms plus variable expiratory airflow limitation at study visit or 2) current asthma symptoms with ongoing daily asthma treatment with inhaled corticosteroids at research visit. Asthma symptoms were defined as wheeze, cough at night or prolonged cough >14 days during airway infections, exercise-induced either chest tightness, wheeze or shortness of breath, or recognition of these symptoms from earlier episodes during the MPT (only if airflow limitation was induced). Allergic asthma was defined as aforementioned plus either aeroallergic sensitisation (>0.35 kU·L^−1^ to any tested specific allergen) or blood eosinophil count >300 cells·µL^−1^. Ever allergy was self-reported and included any aeroallergies or food allergies.

#### Statistical analysis

Due to skewed distributions, data are described with median (interquartile range (IQR)) or number (percentage) of each group. The measures of BHR, PD_20_ and DRS were compared between the viral LRTI and control groups using the Mann–Whitney U-test due to the non-normal distribution of these variables. Additionally, a Kaplan–Meier plot was created to compare the different groups in terms of the proportion of children without BHR over increasing doses of methacholine. The concept of time was replaced with the cumulative dose of methacholine and an event was considered a 20% decline in FEV_1_. The groups were formally compared using Kaplan–Meier statistics. Group comparisons of dichotomous variables were analysed with the Pearson Chi-squared test and Kruskal–Wallis test. Sensitivity analyses for children aged <12 months at exposure were assessed for the main outcomes (lung function, BHR and asthma). Associations for asthma between each specific viral LRTI exposure and controls were assessed by logistic regression. Models were adjusted for available confounders defined *a priori* based on literature and a causal framework, using complete case analyses [26]. Model 1 was adjusted for self-reported individual allergy (yes/no) and parental asthma or allergy (yes/no); model 2 also adjusted for gestational age <36 weeks (yes/no). Results were presented as odds ratios and precision of estimates with 95% confidence intervals. Analyses were done using SPSS version 27.0 (IBM, Armonk, NY, USA) and figures were created in Python (www.python.org).

## Results

### Clinical characteristics of the study population

The distribution of the 135 participants was as follows: 16 MPV, 34 RV, 51 RSV, 13 mixed group and 21 controls. In the MPV group 13 children had single virus infection and three children had viral co-detections (adenovirus, enterovirus, adenovirus and coronavirus OC43). For details about the mixed group, see the supplementary material. At study inclusion during hospitalisation for LRTI, the MPV group was older compared with both the RV and RSV groups (table 1). The MPV group reported the highest proportion of pre-term birth (19%).

**TABLE 1:** Characteristics at hospitalisation for lower respiratory tract infection (LRTI) (n=135, unless otherwise stated)

	**Controls** **(n=21)**	**All viral LRTI** **(n=114)**	**Specific virus groups (n=114)**			
			**MPV (n=16)**	**RV (n=34)**	**RSV (n=51)**	**Mixed (n=13)**
**Age, months**	34 (19–54)	5.8 (2.2–12.7)	10.8 (4.8–12.5)	7.6 (3.0–15.4)	3.0 (1.5–7.8)	13.5 (4.2–17.6)
**Girls**	5 (24)	46 (40)	8 (50)	9 (27)	23 (45)	6 (46)
**Length of hospital stay >24 h, n=114**		94 (83)	12 (75)	28 (82)	45 (88)	9 (69)
**Need for oxygen supplementation, n=114**		57 (50)	8 (50)	16 (47)	26 (50)	7 (54)
**Treatment with oral steroids, n=112**		24 (21)	*2 (13)*	*13 (39)*	*7 (14)*	*2 (15)*
**Respiratory support, n=114**		12 (11)	1 (6)	2 (6)	9 (18)	0
**Any radiological infiltrate, n=68**		51 (75)	9 (82)	12 (67)	28 (85)	2 (33)
**Viral co-detection^#^, n=131**	17 (81)	15 (13)	3 (19)	0	0	12 (92)
***Streptococcus pneumoniae*^#^, n=128**	10 (56)	46 (42)	5 (31)	15 (50)	21 (41)	5 (39)
***Moraxella catarrhalis*^#^, n=128**	8 (44)	41 (37)	7 (44)	15 (50)	15 (29)	4 (31)
***Haemophilus influenzae*^#^, n=128**	4 (22)	38 (35)	9 (56)	13 (43)	13 (25)	3 (23)
**Caesarean section**	8 (38)	24 (21)	5 (31)	3 (9)	13 (26)	3 (23)
**Gestational age <36 weeks**	1 (5)	8 (7)	3 (19)	2 (6)	2 (4)	1 (8)

Data are presented as median (interquartile range) or n (%). MPV: metapneumovirus; RV: rhinovirus; RSV: respiratory syncytial virus. ^#^: nasopharyngeal aspirate at exposure. Viral co-detection is the presence of more than one virus for the exposure group. For the control group viral co-detection is the presence of one or more viruses. The included co-detections do not include RSV or RV in the MPV group. For continuous variables the Mann–Whitney U-test was used and for dichotomous variables the Pearson Chi-squared test was used. Italic values denote p<0.05 for comparison across groups.

At follow-up, the participants’ median (IQR) age was 9.3 (8.3–10.6) years (table 2). The control group reported a higher prevalence of ever airway allergy compared with LRTI children overall (33% *versus* 13%; p=0.02). None of the MPV children reported airway allergy, whereas 15% among RV, 12% among RSV and 31% among the mixed group children reported ever airway allergy (p=0.03).

**TABLE 2:** Self-reported characteristics at follow-up (n=135, unless otherwise stated)

	**Controls** **(n=21)**	**All viral LRTI** **(n=114)**	**Specific virus groups (n=114)**			
			**MPV (n=16)**	**RV (n=34)**	**RSV (n=51)**	**Mixed (n=13)**
**Age, years**	10.5 (9.6–11.4)	**9.2 (8.3–10.1)**	8.9 (8.0–11.1)	8.4 (7.7–9.0)	9.8 (9.0–10.8)	9.1 (7.8–10.5)
**Smoking pregnancy**	0	7 (6)	2 (13)	0	4 (8)	1 (8)
**Parental smoking**	1 (5)	20 (18)	3 (19)	5 (15)	9 (18)	3 (23)
**Breastfeeding >4 months, n=100**	17 (100)	76 (92)	11 (100)	20 (87)	38 (95)	7 (78)
**Parental asthma or atopy**	13 (62)	82 (72)	10 (63)	27 (79)	34 (67)	11 (85)
**Allergen exposure <2 years^#^**	5 (24)	49 (43)	8 (50)	10 (29)	26 (51)	5 (39)
**Ever eczema**	7 (33)	52 (46)	6 (38)	14 (41)	26 (51)	6 (46)
**Ever airway allergy**	7 (33)	**15 (13)**	*0*	*5 (15)*	*6 (12)*	*4 (31)*
**Ever doctor-diagnosed asthma**	4 (19)	33 (29)	6 (38)	10 (29)	13 (26)	4 (31)
**Current asthma controller treatment (ICS or LTRA)**	2 (10)	8 (7)	2 (13)	5 (15)	1 (2)	0
**Current allergy treatment (nasal steroids or antihistamines)**	1 (5)	7 (6)	0	3 (9)	2 (4)	2 (15)

Data are presented as median (interquartile range) or n (%). LRTI: lower respiratory tract infection; MPV: metapneumovirus; RV: rhinovirus; RSV: respiratory syncytial virus; ICS: inhaled corticosteroid; LTRA: leukotriene receptor antagonist. ^#^: living in home with a dog, cat, other furred animal, carpets in living room/bedroom or mould during first 2 years of life. For continuous variables the t-test was used and for dichotomous variables the Pearson Chi-squared test was used. Bold values denote p<0.05 for comparison with controls; italic values denote p<0.05 for comparison across groups.

### Lung function and BHR

**FIGURE 1:**
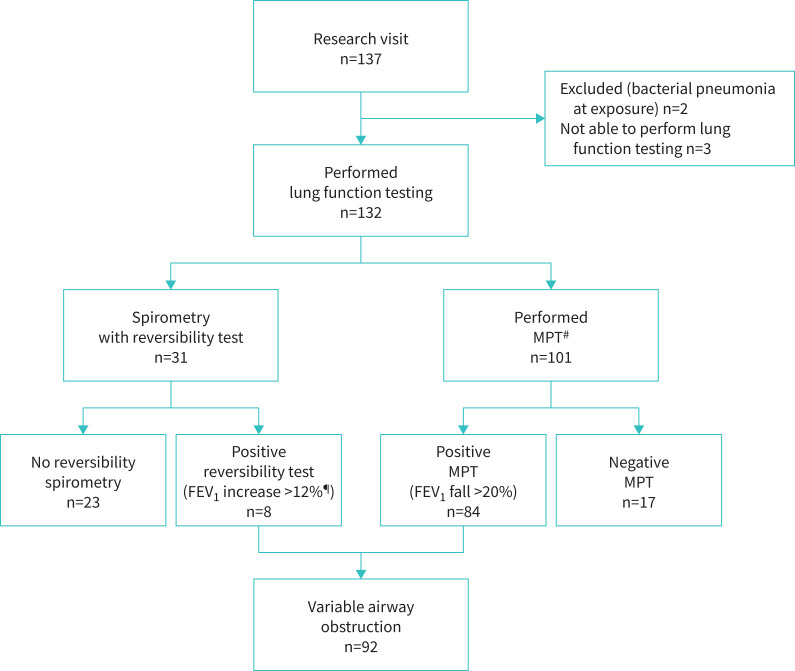
Adherence to the protocol for lung function testing. ^#^: contraindications for the methacholine provocation test (MPT) (n=31): respiratory infection within the last 2 weeks (n=8), inability/unwilling to perform the MPT (n=13) and clinical/spirometric signs of airway obstruction (n=10); ^¶^: after inhalation of 0.4 mg salbutamol. FEV_1_: forced expiratory volume in 1 s.

**FIGURE 2:**
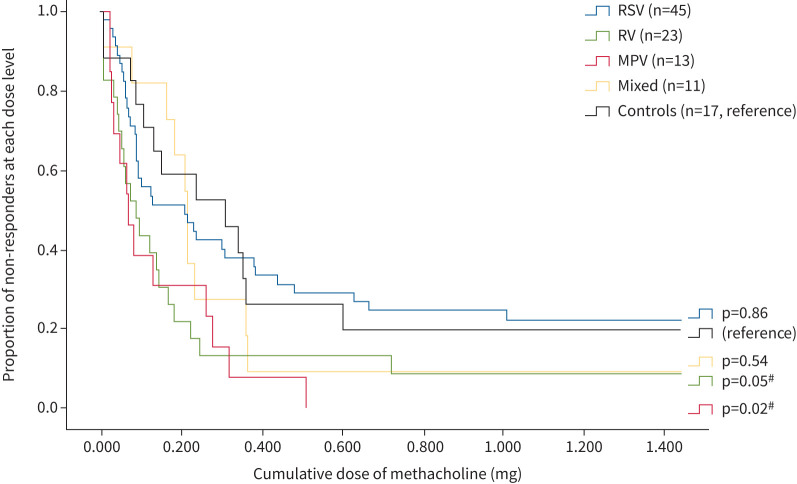
Bronchial hyperresponsiveness in children after specific virus bronchiolitis compared with control children. The censoring dose of methacholine was 1.447 mg. RSV: respiratory syncytial virus; RV: rhinovirus; MPV: metapneumovirus. The virus groups were formally compared pairwise with controls by using a log-rank test. ^#^: p<0.05.

Adherence to the protocol for lung function testing is shown in figure 1. Most children (132 (98%)) were able to perform spirometry and 101 (75%) performed the MPT. The baseline spirometry values did not differ between the groups (table 3). The MPT revealed higher BHR among the virus-infected children (expressed as lower PD_20_ or quantified as higher DRS). Among all LRTI children, PD_20_ was 104 µg compared with 240 µg in the controls (p=0.05). For MPV PD_20_ was 70 µg (p=0.03), for RV 99 µg (p=0.03) and for RSV 95 µg (p=0.10) compared with controls. The LRTI group had a higher DRS compared with controls (136 *versus* 57%·mg^−1^; p=0.03). Considering each viral group, DRS was higher among MPV (DRS 259%·mg^−1^; p<0.01) and RV (DRS 172%·mg^−1^; p=0.01) compared with controls (DRS 57%·mg^−1^). DRS for RSV was 128%·mg^−1^ (p=0.16 compared with controls). The survival plot in figure 2 compares BHR in all children who completed the methacholine challenge or had variable airway obstruction without provocation. Using Kaplan–Meier statistics, BHR was increased for both MPV and RV when compared with controls (p=0.02 and p=0.05, respectively) and when compared with RSV (p=0.01 and p=0.03, respectively). In a sensitivity analysis with only children aged <12 months at exposure for LRTI included, the results showed even more increased BHR for MPV (supplementary table); PD_20_ was 59 µg for MPV compared with 240 µg for controls (p=0.04) and DRS was 368%·mg^−1^ for MPV compared with 57%·mg^−1^ for controls (p=0.01). The corresponding results for the Kaplan–Meier statistics also showed increased BHR for MPV when compared with RSV (p=0.02) and controls (p=0.03). This was not seen for RV nor RSV.

**TABLE 3:** Laboratory findings, lung function and asthma at follow-up (n=135, unless otherwise stated)

	**Controls** **(n=21)**	**All viral LRTI** **(n=114)**	**Specific virus groups (n=114)**			
			**MPV (n=16)**	**RV (n=34)**	**RSV (n=51)**	**Mixed (n=13)**
**Total IgE (kU·L^−1^** **), n=112**	84 (19–223)	51 (16–137)	92 (63–166)	23 (14–136)	37 (13–151)	67 (20–87)
**Sensitisation, n=112**	7 (39)	21 (22)	4 (29)	6 (26)	8 (18)	3 (23)
**Eosinophils >300 µL^−1^, n=110**	3 (17)	15 (16)	2 (14)	4 (17)	6 (14)	3 (23)
**FEV_1_ (z-score), n=132**	0.18 (−0.61–0.63)	−0.01 (−0.51–0.54)	−0.27 (−0.74–0.14)	−0.35 (−0.38–0.45)	0.06 (−0.62–0.87)	0.37 (−0.27–0.54)
**FEV_1_/FVC (z-score), n=132**	−0.77 (−1.54– −0.14)	−0.82 (−1.39– −0.16)	−1.16 (−1.43– −0.34)	−0.75 (−1.20– −0.14)	−0.79 (−1.29– −0.14)	−0.99 (−1.57– −1.29)
**PD_20_ (µg), n=84**	240 (109−357)	**104 (64–238)**	**70 (32–273)**	**99 (58–178)**	95 (64–306)	216 (176–301)
**Dose–response slope (%·mg^−1^), n=101**	57 (34–164)	**136 (54–284)**	**259 (68–662)**	**172 (79–293)**	128 (20–280)	80 (54–233)
**Asthma**	5 (24)	52 (46)	8 (50)	17 (50)	22 (43)	5 (39)
**Allergic asthma**	3 (14)	19 (17)	4 (25)	6 (18)	5 (10)	4 (31)

Data are presented as median (interquartile range) or n (%). LRTI: lower respiratory tract infection; MPV: metapneumovirus; RV: rhinovirus; RSV: respiratory syncytial virus; FEV_1_: forced expiratory volume 1 s; FVC: forced vital capacity; PD_20_: provocative dose of methacholine necessary to produce a fall in FEV_1_ of 20%. For continuous variables the Mann–Whitney U-test was used and for dichotomous variables the Pearson Chi-squared test was used. Bold values denote p<0.05 for comparison with controls.

### Current asthma

**FIGURE 3:**
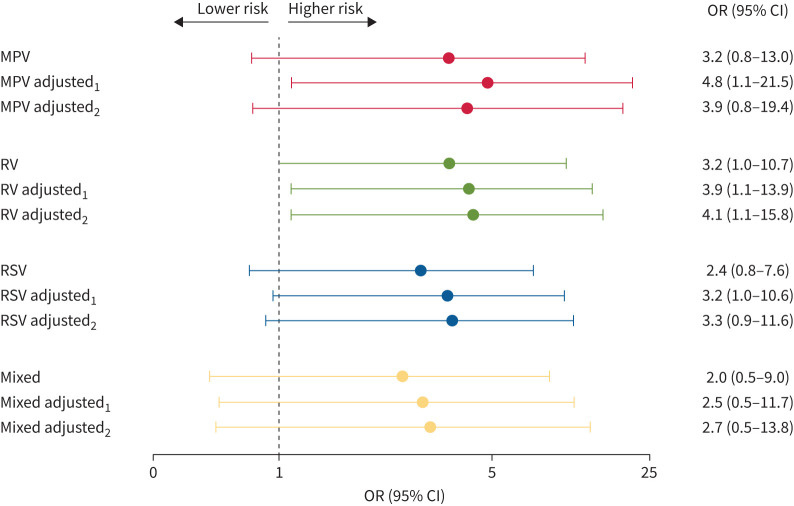
Crude and adjusted odds ratios for asthma after specific virus bronchiolitis compared with controls. Model 1 (adjusted_1_) was adjusted for self-reported allergy and parental asthma or allergy. Model 2 (adjusted_2_) was in addition adjusted for pre-term birth <36 weeks. MPV: metapneumovirus; RV: rhinovirus; RSV: respiratory syncytial virus.

The proportion of children with current asthma at follow-up was higher among LRTI children compared with controls (46% versus 24%; p=0.06) (table 3). Among the 52 children diagnosed with asthma, 47 fulfilled the definition with variable airway obstruction and asthma symptoms, whereas for five children the definition was based on current asthma symptoms and daily use of controller treatment without the possibility for withdrawal. In the MPV and RV groups, 50% fulfilled the asthma criteria, compared with 43% in the RSV group (p=0.37). The prevalence of allergic asthma was 20% among virus-infected children and 15% among controls. Using logistic regression, odds ratios for asthma in the virus groups compared with controls were 3-fold for all viruses (figure 3). Adjustment for parental atopy, history of allergy or pre-term birth did not attenuate the associations.

## Discussion

The main finding in our study was profoundly altered BHR at school age after early-life LRTI with MPV. This was seen also after RV, and to a less degree after RSV, virus groups were compared with control children. We also found a high prevalence of asthma and of allergic asthma in all virus groups. However, relatively small numbers yielded low precision in estimates for asthma and allergic asthma related to specific virus groups.

The main finding with increased BHR after LRTI is in line with Mikalsen *et al.* [27], who found the same in children 11 years of age after bronchiolitis when compared with controls. Their comparison only distinguished between RSV and non-RSV aetiology, where the latter group had higher BHR. Based on studies on prevalence of viral aetiologies in early-life viral LRTI, most non-RSV in that study were likely to have been infections with RV, co-infections or MPV [10–12] and therefore similar to our findings. We found only one study (81 children, 12.3 years median age at follow-up) that assessed BHR (exercise induced or MPT) after bronchiolitis with specified virus aetiology (RV and RSV), but that study found no association [28]. BHR is a hallmark of asthma, and a treatable trait in asthma important to identify in both intervention and epidemiological studies [29]. The use of bronchial provocation tests is recommended in the recent European Respiratory Society Task Force guidelines for the diagnosis of asthma in children [30] and the methacholine provocation in the present study is a clear strength.

We observed a high prevalence of current asthma at school age in all virus groups after LRTI (overall 46%), but also among controls with no history of LRTI (24%). The data indicate a 3–4-fold increased risk for asthma after LRTI with MPV, RV and RSV when compared with controls, but we lacked power for firm conclusions about virus-specific associations. The asthma prevalence was high compared with other early-life infection cohorts, where asthma prevalence varies from 16% to 35% [27, 31]. We found only one follow-up study for asthma after early-life MPV infection specifically; García-García *et al.* [32] found a prevalence of 69% in pre-school children, but the diagnosis was based on clinical interviews. In the present study, allergic asthma was almost twice as common after LRTI with MPV and RV compared with RSV, although small numbers and results should be interpreted with caution. High prevalence of allergic asthma has earlier been shown after RV bronchiolitis [31] and allergic sensitisation has been shown to precede LRTI with RV [33].

Even though the association between early-age LRTI and asthma is well established [6, 7], and there is increasing evidence of between-virus differences [15], there is still lacking evidence for effective prevention strategies for asthma. We still have an incomplete understanding of the abnormal immune response, revealed or mediated by a LRTI, and later asthma development. However, there are experimental studies that support similar responses in RSV and MPV towards T-helper type 2 cells and ineffective viral clearance that in turn may be linked to asthma development [34]. For prevention, there is some evidence that prednisolone after non-RSV bronchiolitis can reduce wheeze during the first 12 months [35]. To design good randomised controlled trials for asthma prevention, it is important to continue defining these risk subgroups presenting with LRTI. Viral aetiology, including MPV, is one risk factor we consider must be part of this prognostic enrichment strategy [36]. Defining LRTI categories by viral aetiology is a single dimension approach and requires high-quality microbial methodology. Our cohort has previously been described [16, 22] and among children diagnosed with bronchiolitis in this project (n=2560) the detection rate of any virus was 95% (unpublished data). Viral co-detections further complicate risk assessment of asthma [37], but this information is not always available in follow-up studies. We have previously identified 37% viral co-detections in children hospitalised with MPV or RSV in our cohort [20]. In our study we selected RSV and RV LRTIs with single virus detection for a stricter definition of viral aetiology. For the less frequent MPV, co-detections other than RV and RSV were permitted, and three of the children had co-detections with viruses not known to be associated with asthma (adenovirus, coronavirus OC43 or enterovirus). The possible clinical role of these co-detections in LRTI is suggested to be low. Another way to approach the association between LRTI and asthma is defining clinical phenotypes. Petrarca *et al.* [38] used data analysis to define homogenous clusters of characteristics within a large cohort of bronchiolitis patients. They found three main profiles with significant differences in associated asthma risks; at 7 years the highest risk was seen among children who presented with elevated eosinophils and RV at exposure [38]. RV was also associated with increased asthma risk after profiling severe bronchiolitis [14]. However, the viral detection rate varies and in these two studies was lacking in 43% and 14%, respectively, of the samples.

The main weaknesses of our study are linked to selection bias and the relatively low sample size. The follow-up included hospital investigation with blood and lung function tests that may have prevented participation, particularly in low-risk children. A lower percentage of invited children (22%) met from the control group than from the virus-infected group overall (41%). The control children reported a relatively high prevalence of both parental atopy and individual allergy, probably due to a stronger selection bias for children with allergy or asthma symptoms in this group. Nevertheless, despite selection bias towards more atopic children in the control group, the LRTI children still showed more BHR and asthma. However, it is unlikely that selection bias differed between specific virus groups. The low prevalence of MPV among LRTI children <2 years of age resulted in a low sample size and limited the statistical power of the study.

The main strengths of our study were the prospective design with high-quality and detailed microbial exposure data, and the state-of-the-art lung function tests, including reversibility and bronchial provocation with methacholine [30].

In conclusion, we found increased BHR and a high prevalence of asthma in school-aged children after early-life MPV infection, and findings were similar after RV. The findings support increasing evidence that virus aetiology in LRTI matters to predict, or potentially change, asthma risk. We call for more studies on long-term respiratory outcomes after early-life MPV infection and the factors that could mediate these associations.

## Supplementary material

10.1183/23120541.00832-2023.Supp1 **Please note:** supplementary material is not edited by the Editorial Office, and is uploaded as it has been supplied by the author.
Supplementary material 00832-2023.SUPPLEMENT
