# Supplementary material for: Bronchial reactivity and asthma at school age after early-life metapneumovirus infection
Source: ERJ Open Res. 2024 Jan 22;10(1):00832-2023. doi: 10.1183/23120541.00832-2023 (PMC10801746; doi:10.1183/23120541.00832-2023)
Supplement: Supplementary file 1 [file 00832-2023.SUPPLEMENT.pdf]

Supplementary online material:

Methods:

Exposure: The following viruses were analysed in both LRT exposed and control children's samples: RSV, RV, MPV, BoV, 4 subtypes of coronavirus, human adenovirus, human enterovirus, human parechovirus, influenza virus A and B, 4 subtypes parainfluenza virus. Details on NPA tests have been described earlier [1]. Chest radiographs were done on clinical indication.

Definitions:

Allergen exposure was defined as living in home with at least one of the following exposures during the two first years of life; dog, cat, other furry animal, carpets livingroom/bedroom or mold. Respiratory support was defined as the need of either continuous positive airway pressure (CPAP), biphasic positive airway pressure (BIPAP) or invasive mechanical ventilation.

Laboratory testing:

Leukocytes and a differential blood count were analyzed. Total IgE and allergy panels for aeroallergens (ImmunoCAP Phadiatop) and food allergens (ImmunoCAP Allergen fx5) were analyzed (Thermo Fischer Scientific, Norway). Reflex testing of specific allergens was done if initial panel testing was positive; aeroallergens: g6 (timothy), t3 (birch), t9 (olive), e1 (cat dander), e3 (horse dander), e5 (dog dander), w6 (mugwort), w19 (Parietaria officinalis), d1 (Dermatophagoides pteronyssinus), d2 (Dermatophagoides farina), m2 (Cladosporium herbarum) and 6 food allergens: f1 (egg white), f2 (milk), f3 (cod), f4 (wheat), and f13 (peanut) and f14 (soybean).

Lung function tests:

Lung function was measured according to established guidelines using a spirometer including a module for bronchial provocation tests (Vyntus APS Pneumo EU, AkuMed, Norway). The results were expressed as z-scores according to the European Respiratory Society Global Lung Function Initiative [2]. After initial baseline flow-volume spirometry all children were considered for a methacholine provocation test (MPT) to measure eventual bronchial hyperresponsiveness. Contraindications for the MPT was any airway infection, ongoing antibiotic treatment, or asthma exacerbation during the last two weeks, FEV_1 <70% (forced expiratory volume in one second), clinical signs of airway obstruction or technical inability to perform reproducible spirometry. The MPT was performed with an inhalation-synchronized dosimetry nebulizer. The test procedure implicated doubling of methacholine doses until a 20% fall in FEV_1 or to a maximum cumulative dose of 1.447 mg methacholine. All children concluded the lung function testing with a spirometry 10 minutes after inhalation of salbutamol 0,4 mg (Ventoline®) through a spacer (OptiChamber Diamond® by Philips Respironics).

Results:

Study population:

A total of 460 children from the original bronchiolitis cohort and their controls were identified for follow up, of which 90 were excluded because of travel time. An invitation letter was sent to 370 children and 137 (37%) attended the follow-up. Participation according to virus group was distributed as follows (% of invited) 16 MPV (33%), 34 RV (37%), 51 RSV (50%), 13 mixed group (37%) and 21 controls

(23%). In the mixed group there were 3 children with co-detection of RV and RSV; 1 with RV, RSV and cytomegalovirus; 1 with RV, RSV and varicella; 1 with RV and MPV; 1 with RSV and BoV; 1 with RV and BoV; 1 with MPV, BoV and adenovirus; 1 with BoV and adenovirus; 1 with BoV and coronavirus C229e; 1 with BoV and coronavirus OC43; and 1 with BoV and parechovirus.

	Controls<br>n=21	All viral LRTI<br>n=80	Specific virus groups<br>n=80			
			**MPV**<br>**n=11**	**RV**<br>**n=18**	**RSV**<br>**n=45**	**Mixed**<br>**n=6**
**FEV1 (z-score)**<br>**N=98**	0.18<br>(-0.61, 0.63)	0.13<br>(-0.47, 0.56)	-0.29<br>(-0.76, 0.45)	0.17<br>(-0.27, 0.49)	0.06<br>(-0.57, 0.80)	0.37<br>(0.18, 1.50)
**FEV1/FVC (z-score)**<br>**N=98**	-0.77<br>(-1.54, -0.14)	-0.79<br>(-1.30, -0.14)	-1.28<br>(-1.46, -0.27)	-0.88<br>(-1.20, -0.70)	-0.73<br>(-1.27, -0.10)	-0.85<br>(-1.51, 0.14)
**PD20 (μg)**<br>**N=63**	240<br>(109 –357)	115<br>(62 – 247)	**59***<br>(28 - 280)	125<br>(64 – 171)	95<br>(64 – 311)	225<br>(178 – 333)
**Dose Response Slope (% /mg)**<br>**N=77**	57<br>(34 – 164)	**136***<br>(43 - 293)	**368***<br>(70 – 816)	148<br>(52 – 279)	145<br>(29 - 282)	79<br>(57 – 117)
**Asthma, n (%)**	5 (24)	35 (44)	6 (55)	9 (50)	18 (40)	2 (34)
**Allergic asthma, n (%)**	3 (14)	13 (16)	4 (36)	3 (17)	4 (9)	4 (33)

Supplementary table: Lung function and asthma in children < 12 months at exposure

Abbreviations: LRTI (lower respiratory tract infection). MPV (metapneumovirus). RV (rhinovirus). RSV (respiratory syncytial virus). FEV1 (forced expiratory volume 1 second). FVC (forced vital capacity). PD20 (provocation dose). Data are presented as medians (interquartile range) unless otherwise stated. For continuous variables, the Mann Whitney U test was used and for dichotomous variables Pearson's chi- square test. Bold values denote p ≤0.05. *Comparison with controls. N=101 unless otherwise stated.

1. Heimdal I, Lysvand H, Krokstad S, et al., Detection of subgenomic mRNA from endemic human coronavirus OC43 and NL63 compared to viral genomic loads, single virus detection and clinical manifestations in children with respiratory tract infections. J. Clin. Virol., 2022. 154: p. 105247.

2. Quanjer PH, Stanojevic S, Cole TJ, et al., Multi-ethnic reference values for spirometry for the 3-95-yr age range: the global lung function 2012 equations. Eur. Respir. J., 2012. 40(6): p. 1324-1343.
